# Knowledge, attitude and practice of influenza vaccination among Lebanese parents: A cross-sectional survey from a developing country

**DOI:** 10.1371/journal.pone.0258258

**Published:** 2021-10-14

**Authors:** Ramia Zakhour, Hani Tamim, Farah Faytrouni, Joanne Khoury, Maha Makki, Lama Charafeddine

**Affiliations:** 1 Department of Pediatrics and Adolescent Medicine, American University of Beirut Medical Center, Beirut, Lebanon; 2 Department of Internal Medicine, Clinical Research Institute, American University of Beirut Medical Center, Beirut, Lebanon; 3 Clinical Research Institute, American University of Beirut Medical Center, Beirut, Lebanon; University of Sharjah College of Health Sciences, UNITED ARAB EMIRATES

## Abstract

**Background:**

A growing number of parents refuse vaccination due to concerns about side effects. Influenza vaccine is no exception and remains one of the most controversial vaccines. Data regarding influenza vaccine uptake and parental knowledge, attitude and practice towards vaccination in the Lebanese population is lacking. The aim of this study was to assess the rate of vaccination refusal and potential associated factors among Lebanese parents of school-aged children, in general and with a focus on influenza vaccine.

**Methods:**

A parent questionnaire was distributed in randomly selected 2 public and 2 private schools from the greater Beirut area during the school year 2017–2018. Questionnaires covered knowledge, attitude (including themes of efficacy, hesitancy and trust), and practice of vaccination in general and influenza vaccine in particular.

**Results:**

The response rate was 76.5% (306/400). Overall, 29.4% parents reported vaccinating their children against influenza (62.2% in private and 37.7% in public schools). Younger age, paternal employment and higher household income were associated with higher vaccination rates (p = 0.01, 0.02 and <0.0001 respectively). Lack of vaccine recommendation by the physician was the most common reason for not taking it (47%). Parents who accepted influenza vaccination had higher scores in efficacy, hesitancy and trust and were more compliant with other vaccinations.

**Conclusion:**

One third of parents of school aged children in the greater Beirut area vaccinate their children against influenza. This rate is likely lower in rural remote areas. Physician’s recommendation is the single most important predictor of such vaccination. Future studies tackling physicians’ attitude and practice are needed to help improve influenza vaccination rates in the Lebanese population.

## Introduction

The world is facing the resurgence of multiple outbreaks of vaccine preventable diseases that endanger children’s health [1]. A major cause is the falling vaccination rates and the blooming anti-vaccination campaigns [2]. The most notorious example is the anti-measles movement founded in 1998 after a fraudulent paper linking the mumps measles rubella (MMR) vaccine to autism [3]. This fallacy remains a source of concern and anxiety to many parents to this date despite the discreditation of the original paper. Physicians continue to encounter daily “hesitant” families, who argue against the need for immunization.

This trend of anti-vaccines has been expanding to reach the middle-income countries, and many Arab countries [4]. Influenza vaccine is one of the vaccines that generate many controversies among parents, despite the fact that influenza infection is associated with significant morbidity and mortality, especially in young children. The World Health organization (WHO) estimates that influenza epidemic results in approximately 3 to 5 million cases every year of severe illness with secondary complications and around 250,000 to 500,000 deaths reported globally, affecting most frequently very young children, elderly population and patients with other comorbidities [5]. Although influenza vaccine reduces overall health care cost and is recognized as the most effective measure for preventing influenza infection, coverage rates are still suboptimal worldwide.

Since 2008, the Advisory Committee on Immunization Practices (ACIP) recommends yearly influenza vaccination to all children 6 months and older. However, in the 2017–2018 influenza season, only 57.9% of children between the ages 6 months and 17 years were vaccinated in the United States (United States) [6]. According to the CDC, 186 pediatric deaths attributed to influenza were reported during that season and 80% of these cases had not received the influenza vaccine. Data from Japan estimated that only 11% of children younger than one year of age, 70% of children 1 to 6 years of age and 58% of children 6 to 13 years of age were vaccinated against influenza in the 2010–2011 season [7]. In England, 52.8% of children age 2–7 years were reported to have received the influenza vaccine for the 2015–2016 season [8], and in Australia, the number of vaccinated children decreased from 42% in 2008–2009 to 7.1% in 2010–2014 [9].

Some of the barriers to influenza vaccines uptake described in the literature relate to poor socio-economic status, concerns about vaccine side effects, safety, effectiveness, and the high number of vaccines that children receive [8]. Other reasons relate to parental belief that influenza vaccine might actually cause the disease, lack of awareness about the seriousness of the diseases or lack of recommendation from physician, in addition to vaccine accessibility and financial barriers [9, 10].

In Lebanon, influenza vaccine is not part of the national vaccination program; as such, it is not mandatory and is not funded by the ministry of public health. A hospital-based surveillance system for influenza currently exists in Lebanon through the ministry of public health (MOPH) however clinic-based surveillance does not exist, neither does a burden of disease or a vaccine uptake/coverage database.

In the Middle East and North Africa (MENA) region, eight studies addressed parental knowledge and attitude regarding childhood immunization and their impact on the decision-making process (Egypt [11], Iraq [12], UAE [13], KSA [14] and Jordan [15]), however none focused on influenza vaccine. In Lebanon, limited information is available as the only studies available do not report vaccination practice among children [16].

The aim of this study is to describe attitudes, knowledge and practice of Lebanese parents in regard to vaccination in general with a focus on specific vaccines. This paper will focus on findings related to the influenza vaccine.

## Material and methods

### Study population data collection

This was a cross-sectional survey conducted between November and December 2017 (school year 2017–2018) in a random sample of public and private schools in greater Beirut. A questionnaire addressing knowledge, attitude and practice of vaccination was developed and distributed to parents of children aged 3 to 18 years.

All Lebanese parents of school aged children between 3–18 years and enrolled in the chosen schools were invited to participate. Non-Lebanese parents and parents who were less than 18 years old were excluded.

The list of all private and public schools in the greater Beirut area was obtained from the Lebanese ministry of Education. Four schools (two private and two public) were randomly chosen using a computer-generated sequence. The schools were chosen from two different districts in Beirut to capture all socio-demographic levels. The study was limited to the greater Beirut area for feasibility purposes, although this is not representative of the entire Lebanese population, Beirut is the largest urban conglomeration in the country and hosts almost half of the country’s population including people originally from different regions. A target sample size of 400 was chosen based on logistics and convenience and seemed to be reasonable upon reviewing other similar studies [11, 14]. Survey questionnaires were distributed to all eligible students enrolled in the 4 schools; with the expectation to have 100 questionnaires completed and returned per school. The research team distributed and collected the questionnaires from the schools two weeks after its destination. To protect privacy and data confidentiality, students received the anonymous questionnaire in a sealed envelope along with a letter addressed to parents informing them about the study.

Approvals were secured from the ministry of public health and the respective schools’ administrations to conduct this study after obtaining the university’s Institutional Review Board approval. Oral consents were secured from parents before filling the questionnaire. The consent was on the first page of the survey. It was mentioned in the informed consent that by completing and submitting this survey, participants are indicating that they consent to participate in the study. The institutional review board guaranteed exemption from written consent.

### Survey and scoring of questions

After reviewing related surveys in the literature, a questionnaire was developed and adapted to the local culture and context in Arabic and English by the authors (LC, FF) [15, 17, 18]. The final questionnaire consisted of 50 questions divided into four sections: knowledge, attitude, practice regarding childhood vaccines, and socio-demographic data (age, education, occupation, etc) (S1 Appendix). The questionnaire was first piloted on 15 parents recruited from different socioeconomic levels, asking about its clarity, comprehension, length and cultural acceptability. No modifications were required after piloting. The piloted questionnaires were not included in the analysis.

For each of the knowledge and attitude sections, questions were grouped into themes. For knowledge 3 themes were identified: efficacy (4 questions asking whether they thought vaccines were effective in protecting from diseases, important for the community and improve the immune system), safety (11 questions about vaccine risks and benefits, side effects, harm and long term consequences such as development of autism, diabetes, learning disability, sudden infant death) and general knowledge (6 questions about vaccines in general whether they are for children only, needed only for certain diseases but not others, vaccine schedule and whether they are getting enough information about vaccines and their safety). (Table 1 in S2 Appendix). For attitude, 3 themes were included: reasons (3 questions including whether giving vaccine is only to enter daycare or school, they think there is another was to protect their child and whether they knew someone who did not give vaccine to their child), trust (6 questions about trusting the information received and the ministry of public health vaccine schedule, feeling satisfied with the vaccine delivery, and the information given by the doctor and whether they recommend vaccination to others), and hesitancy (5 questions asking about reluctance to vaccines, concerns regarding side effects, hesitance bout new vaccines and vaccines in general) (Table 2 in S2 Appendix). There were three questions related to practice and behavior asking about having given or not the influenza vaccine and the reason for refusing (Table 3 in S2 Appendix). For each question of the knowledge and attitude sections the answer was converted into a score over 100. A score over 100 was calculated for each theme which was equal to the average score of individual questions under that theme. Basically, questions had 5 point Likert-scale, questions with positive answers were given a score of 100 for strongly agree and 0 for strongly disagree (the scores in between were 25, 50 & 75), the inverse was done for questions with negative answers.

### Statistical analysis

Data in S1 File was managed using the IBM-Statistical Package for Social Sciences (SPSS) version 23. Descriptive statistics are reported with categorical variables as absolute and relative frequencies, continuous variables are described in means and standard deviations. The association between influenza vaccine and other categorical variables was assessed using the Chi-square test, whereas Student’s t-test was used for the association with continuous variables. A p-value of <0.05 indicates statistical significance.

## Results

### Demographics

The sociodemographic characteristics of the total participants and for public and private schools separately are shown in Table 1 and Table 1 in S3 Appendix respectively. Out of 400 questionnaires distributed: 306 parents returned the questionnaire (overall response rate was 76.5%; [86% (172) from private schools and 67% (134) from public schools]. The greatest proportion of respondents were parents of children in middle school (p-value = 0.01). Of the 306 respondents, 216 (70.6%) did not vaccinate their children against influenza. Vaccinated children were younger, with a mean age of 10.52 ± 3.61 years, compared to those who were not vaccinated, with a mean age of 12.03 ± 3.09 (p-value = 0.01). Father’s employment and higher household income were associated with a higher proportion of influenza vaccination, with p = 0.02 and p<0.0001 respectively. Most mothers were between the ages 30–50, not employed and had a university or college degree, with none of these factors having a statistically significant effect on influenza vaccination rates. Similar results were noted for father’s age and educational level with no significant effect on vaccination status either.

**Table 1:** Demographic characteristics in relation to influenza vaccination.

	NO (N = 216) No. (%)	YES (N = 90) No. (%)	p-value
**Child Age**	12.03 ± 3.09*	10.52 ± 3.61*	0.001
**School Grade**			0.01
Preschool	11 (5.3)	16 (17.8)	
Elementary School	65 (31.3)	27 (30.0)	
Middle School	114 (54.8)	42 (46.7)	
Secondary School	18 (8.7)	5 (5.6)	
**Parent filling the questionnaire,**			0.51
Father	38 (18.7)	13 (15.5)	
Mother	165 (81.3)	71 (84.5)	
**Mother Age,**			0.22
18–20	2 (0.9)	1 (1.1)	
20–30	6 (2.8)	7 (8.0)	
30–50	193 (91.0)	76 (86.4)	
>50	11 (5.2)	4 (4.5)	
**Mother’s employment,**			0.68
Employed	83 (39.3)	38 (44.2)	
Self-Employed	23 (10.9)	10 (11.6)	
Not Employed	105 (49.8)	38 (44.2)	
**Mother’s Education, n (%)**			0.15
No Formal Schooling	5 (2.4)	4 (4.7)	
Less than High School	42 (19.9)	15 (17.6)	
High School Graduate	48 (22.7)	11 (12.9)	
Technical School/Graduate	18 (8.5)	5 (5.9)	
University/College	98 (46.4)	50 (58.8)	
**Father’s Age, n(%)**			0.62
18–20	1 (0.5)	0 (0.0)	
20–30	2 (1.0)	0 (0.0)	
30–50	149 (72.3)	68 (79.1)	
>50	54 (26.2)	18 (20.9)	
**Father’s Education, n (%)**			0.56
No Formal Schooling	9 (4.3)	4 (4.8)	
Less than High School	55 (26.4)	16 (19.0)	
High School Graduate	40 (19.2)	19 (22.6)	
Technical School Graduate	16 (7.7)	4 (4.8)	
University/College	88(42.3)	41(48.8)	
**Father’s Employment, n (%)**			0.02
Employed	134 (65.4)	46 (53.5)	
Self-Employed	65 (31.7)	40 (46.5)	
Not Employed	6 (2.9)	0 (0.0)	
**Household Income, n (%)**			<0.0001
<1000$/month	59 (38.6)	18 (26.5)	
1000–5000$/month	80 (52.3)	29 (42.6)	
>5000$/month	14 (9.2)	21 (30.9)	

*Mean ± Standard deviation (Range).

### Knowledge

Table 2 summarizes results pertaining to parental knowledge regarding immunizations in general and influenza vaccine in particular, presented by influenza vaccination status. In total, 90 out of 306 respondents (29.4%) reported having given their children the influenza vaccine [(62.2%) in private and (37.7%) in public)]. There were 4 questions around the theme of efficacy, with 2 showing significant results: most parents of children who received the influenza vaccine in private schools (94.44±10.49) believed that vaccines were effective in protecting children from serious illnesses compared to those who did not received the vaccine (87.5± 17.36, p-value = 0.002). Mostly, parents believed that vaccines are important for protecting the health of others in the community (91.51±16.94 vs 81.64±22.41, p-value = 0.002). Of the surveys that were collected from private schools, 40 / 56 (72.7%) of respondents that gave their children the influenza vaccine believed that there exists a financial barrier to vaccination vs. 62 out of 116 (54.9%) of those who did not receive the vaccine, with a significant p-value = 0.03. While in public schools, 62 out of 100 (63.3%) of those who did not receive the influenza vaccine believed that there exists a financial barrier to vaccination (Table 2 in S3 Appendix). The majority of respondents from both private and public schools reported that their primary source of awareness about vaccination was their doctor (Fig 3A. Those who did not receive the influenza vaccine were more likely to state SMS as a good method of raising vaccination awareness versus those who had received the vaccine (p-value = 0.02).

**Table 2:** Parental knowledge regarding Immunization and its relation to influenza vaccine.

	NO (N = 216) No.(%)	YES (N = 90) No.(%)	p-value
**Barriers**			
Lack of awareness	126 (59.7)	53 (60.2)	0.93
Financial	124 (58.8)	54 (61.4)	0.68
No barriers	34 (16.1)	12 (13.6)	0.59
**Awareness**			
**Source of information**			
Doctor	201 (95.3)	87 (97.8)	0.52
TV	41 (19.4)	15 (16.7)	0.57
Internet	48 (22.7)	24 (26.7)	0.47
School	36 (17.1)	8 (8.9)	0.07
**Best way to raise awareness**			
Group meeting	90 (42.7)	45 (50.0)	0.24
Pamphlets	88 (41.9)	42 (46.7)	0.45
Internet	62 (29.4)	24 (26.7)	0.63
SMS	51 (24.2)	11 (12.2)	**0.02**
TV	92 (43.6)	30 (33.3)	0.10
Doctor	116 (55.0)	62 (68.9)	**0.03**
**Safety scores**	62.51 ± 16.76*	67.91 ± 17.87*	0.01
**Vaccines may cause**			
a. Learning disabilities	69.43 ± 21.76*	76.19 ± 24.82*	0.02
b. Autism	69.53 ± 2.94*	75.89 ± 24.37*	0.04
c. Diabetes	70.29 ± 21.55*	76.19 ± 23.57*	0.04
d. Sudden infant death syndrome	67.80 ± 22.88*	75.00 ± 23.61*	0.02
e. Other chronic diseases	67.45 ± 23.34*	73.51 ± 24.65*	0.05
**Efficacy score**	74.12 ± 13.17*	76.92 ± 11.47*	0.08
**Q1** Childhood vaccines are effective in protecting my child from serious disease.	89.76 ± 15.54*	93.75 ± 10.89*	0.01
**Q2** Having my child vaccinated is important for the health of others in my community	81.94 ± 21.92*	87.07 ± 18.22*	0.055
**Q9** Vaccines are given to children to prevent diseases that are not serious.	46.55 ± 30.59*	44.94 ± 31.35*	0.68
**Q10** Vaccines make the immune system stronger	78.81 ± 19.19*	82.86 ± 18.31*	0.09

*Mean ± Standard deviation (Range).

**Fig 1:**
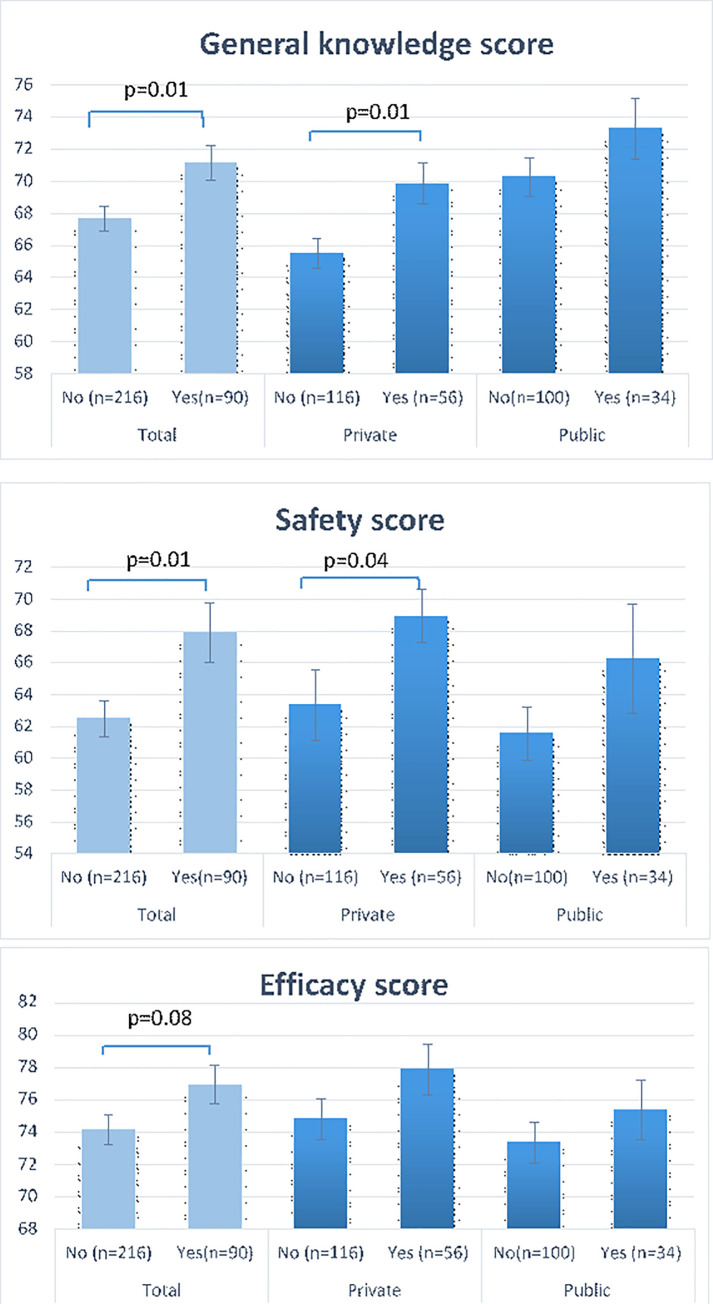
Mean knowledge scores by acceptance of influenza vaccine in private and public schools.

For the questions regarding vaccines being the cause of learning disabilities, autism, diabetes and sudden infant death syndrome, there was a significant difference between those who vaccinated their children versus those who did not (Table 2). Scores of safety, efficacy and general knowledge are displayed in Fig 1. Overall, parents who vaccinated their children against influenza scored higher for safety score of 67.91±17.87 vs those who did not 62.51±16.76 (p = 0.01). The difference was mostly in respondents from private schools rather than public (Fig 1B). The general knowledge scores were higher for those vaccinated 71.14 vs. 67.68 (p = 0.01) and this difference was statistically significant for those in private schools (69.85 vs. 65.5 p = 0.01) rather than public 73.26 vs. 70.24 (p = 0.19) (Fig 1A).

### General attitude and trust

**Fig 2:**
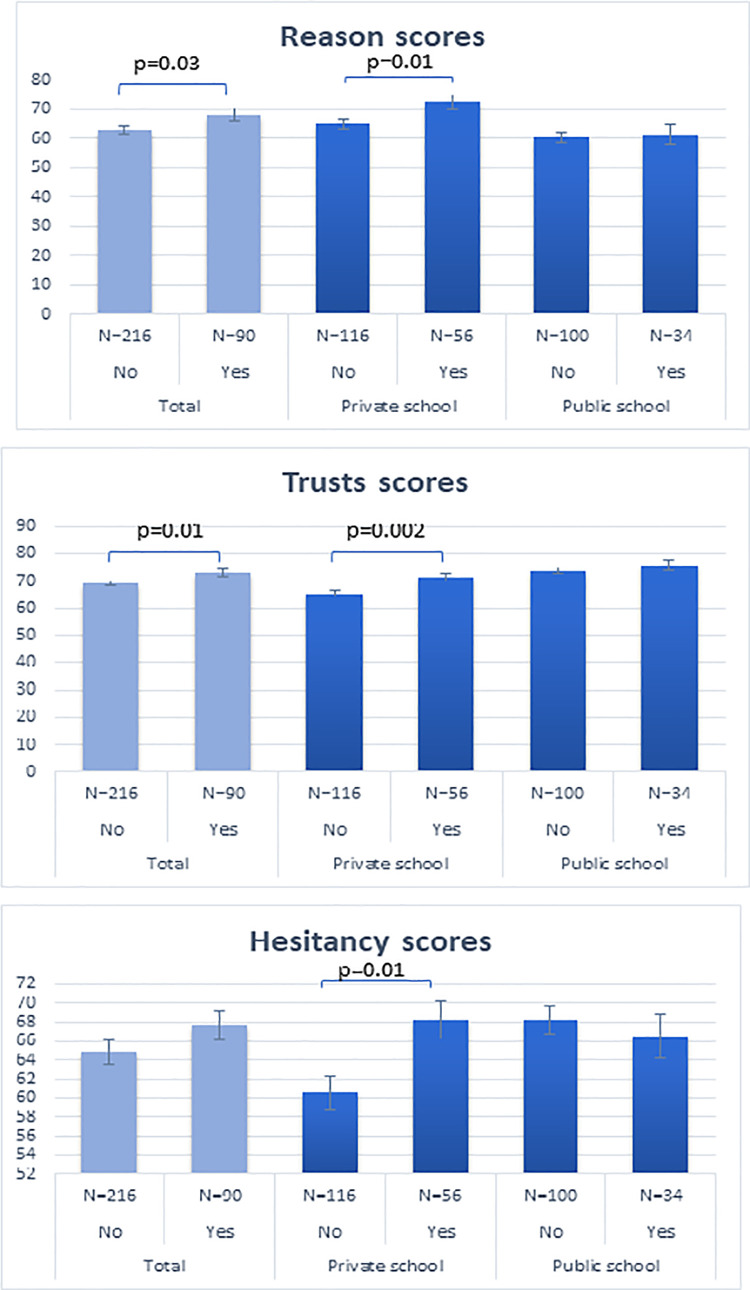
Mean attitude scores by acceptance of influenza vaccine in private and public schools.

Attitude was summarized into three main themes, reasons (3 questions), trust (6 questions) and hesitancy (5 questions): scores by themes are shown in Fig 2.

Overall, mean scores for reason were higher in those vaccinated 68.38 ± 19.28 vs 62.95 ± 19.08 (p = 0.03) and this was mostly due to respondents from private schools. The response of parents in private schools for the reasons questions, were significantly different between those who vaccinated versus those who did not vaccinate their children against influenza 72.62 ± 18.10 vs 65.09 ± 17.91 respectively (p = 0.01); this was not the case for parents from public schools (61.40 ± 19.39 vs 60.42 ± 20.18 (p = 0.81)(Fig 2A).

Similarly, mean scores of trust were higher for those vaccinated vs non-vaccinated (73.03 ± 11.05 vs. 69.19 ± 12.01, p-value = 0.01). This was due to a significant difference seen in mean scores for private school respondents (71.45 ± 11.20 vs 65.41 ± 12.08 p-value = 0.002) (Fig 2B). The mean scores of hesitancy were significantly higher for those who accepted taking the flu vaccine among the private school group (68.20 ± 14.80 vs 60.62 ± 18.73,p = 0.01) (Fig 2C). Table 3 shows the association between attitude, trust and the choice of giving influenza vaccine. In private schools, those who vaccinated their children against influenza generally were satisfied with their physician’s answers about immunizations, followed their physician recommendations and were more likely to recommend immunization to others (Table 3 in S3 Appendix).

**Table 3:** Association between attitude, trust and influenza vaccine.

	No N = 216(%)	Yes N = 90 (%)	p-value
**Perception of knowledge**			
Willingness to give recommended shots	176 (94.1)	81 (96.4)	0.56
Concerns regarding vaccines	167 (86.5)	71 (84.5)	0.66
**Number of concomitant injections considered acceptable**			0.46
1 to 2	86 (41.1)	31 (34.4)	
3 to 4	10 (4.8)	5 (5.6)	
More than 4	7 (3.3)	1 (1.1)	
Whatever the doctor recommends	106 (50.7)	53 (58.9)	
**Concerns of the effects of vaccines**			
Fever	184 (88.0)	77 (86.5)	0.71
Rash	52 (24.9)	19 (21.3)	0.51
Diarrhea	52 (24.9)	12 (13.5)	0.03
Infection	88 (42.1)	34 (38.2)	0.53
Too numerous	49 (23.4)	15 (16.9)	0.20
Do not prevent disease	38 (18.3)	16 (18.0)	0.95
Side effects	90 (43.3)	42 (47.2)	0.53
No concern	80 (38.5)	37 (41.6)	0.61
**Trusts scores**	69.19 ± 12.01	73.03 ± 11.05	0.01
Physicians answers about immunization	77.35 ± 16.66	82.22 ± 18.45	0.03
Physicians recommendations	80.45 ± 18.25	87.36 ± 16.56	0.003
Recommend immunizations to others	86.00 ± 24.68	91.11 ± 17.17	0.04

### Practice and behavior

**Fig 3:**
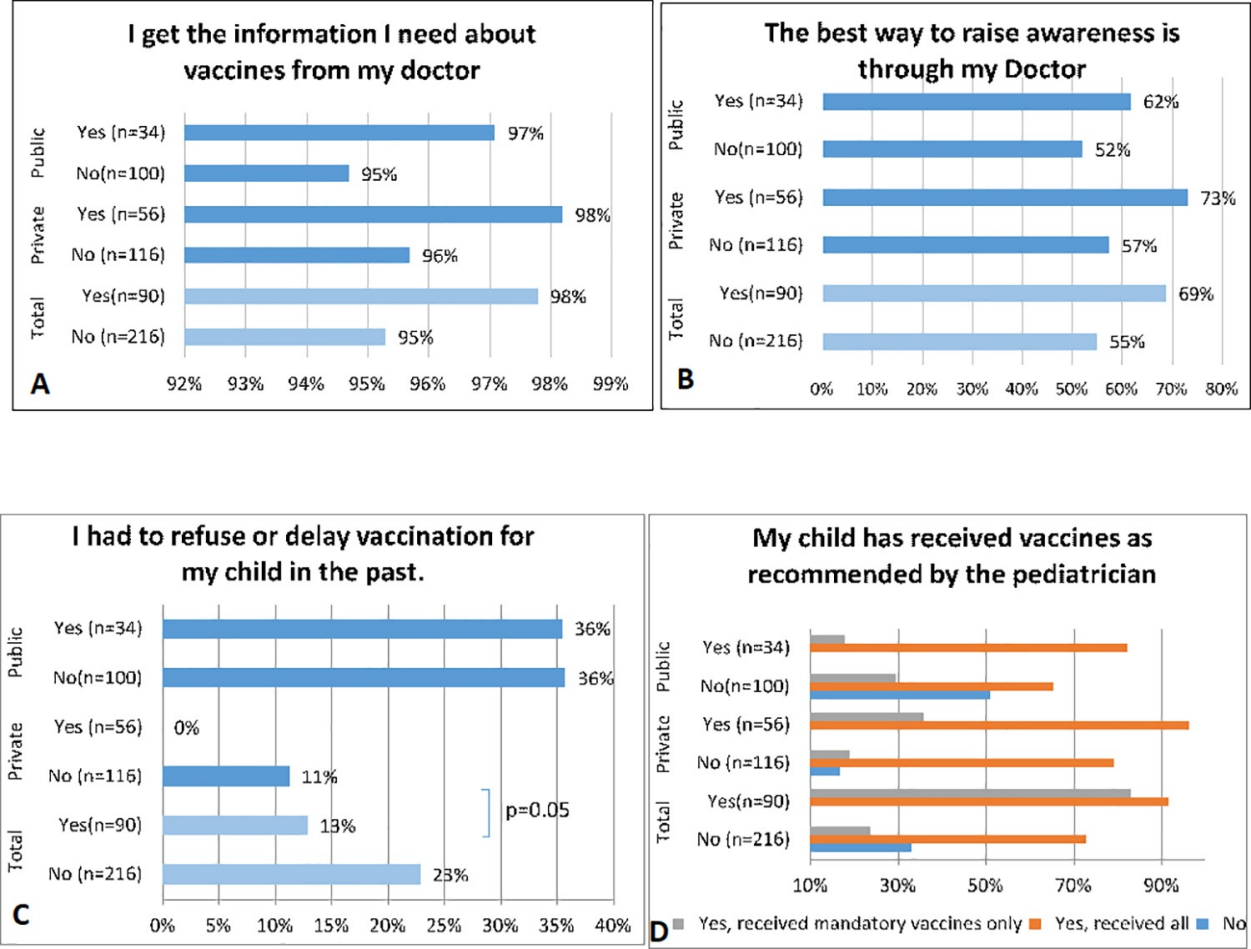
Association between practice behaviors and influenza vaccine.

None of the respondents in private schools who vaccinated their children against influenza delayed or refused vaccination in the past, versus 12 out of 116 (11.3%) of those who did not vaccinate their children (p-value = 0.01) (Fig 3C). Overall, 77 out of 90 (91.7%) of those who administered the influenza vaccine to their children also gave all recommended vaccines compared to 72% of those who don’t give the influenza vaccine 156 out of 216 (p-value = 0.001) (Fig 3D).

Reasons for lack of administration of influenza vaccine are summarized in Table 4. Parents who did not administer the influenza vaccine to their children stated lack of recommendation by their doctor as the most common reason (47.2%). 5.1% stated that they had missed the timeframe for giving it, 5.6% stated that it was too expensive at the time, 14.8% stated that they were hesitant because it might actually cause the “flu” and 10.6% believed that getting influenza infection is not fatal.

**Table 4:** Distribution of reasons for not giving influenza vaccine.

Reason	N (%)
Missed the timeframe for giving it	11 (5.1)
It was too expensive at the time	12 (5.6)
Was hesitant and feared it might actually cause the flu	32 (14.8)
It was not recommended by my doctor	102 (47.2)
Getting the flu is not fatal	23 (10.6)
Other:	
Different strains	3 (18.8)
My son has high immunity	2 (12.5)
Natural immunity	1 (6.3)
Not effective	7 (43.8)
Too much mercury	1 (6.3)
No answer	2 (12.5)

Data available for 216 respondents.

## Discussion

Our study showed that around one third of Lebanese parents vaccinate their children against influenza: rates were higher in private schools compared to public schools. Children in the vaccinated group were overall younger and more likely to have a higher household income. Most respondents (more than 90%) reported their doctors to be the primary source of information regarding vaccines across all categories. Doctor’s office and SMS were the most commonly stated best ways to raise awareness about vaccination. Some of these observations held true in the private school group but not the public school group. This might be due to public schools having a more homogeneous population (or smaller number of vaccinated individuals in the public schools group leading to lower statistical significance of any noted difference). We opted to do subgroup analysis by type of school (public versus private) because in Lebanon there is a clear discrepancy in socioeconomic background of families attending private versus public schools. However the main objective of this study was to investigate the impact of general knowledge, attitude and practice towards vaccine on acceptance of the influenza vaccine and compare knowledge, attitude and practice between groups in favor versus not in favor of the influenza vaccine, the study was not designed to compare knowledge, attitude and practice between private and public schools, although this would be interesting to look at in a future study based on our findings.

Parents who administered the influenza vaccine to their children were likely to believe in vaccine efficacy, have higher trust and display less hesitancy towards vaccines in general. They were less likely to delay vaccination and more likely to give their children all recommended vaccines. Lack of recommendation by physician was the most commonly stated cause for absence of vaccination in the unvaccinated group. A comparable proportion of respondents answered that doctors are their primary source of information regarding vaccines across all groups and in the public school group a good percentage reported following physician recommendations regarding vaccines suggesting that perhaps physicians are not recommending the influenza vaccine frequently or strongly enough in that population.

Since the beginning of 2019 influenza cases have accounted for 33% of severe acute respiratory infection cases that have been reported and tested by the MOPH from 8 sentinel sites across Lebanon. A 2018 review of influenza surveillance and vaccination in the MENA region reported that out of 518 analyzed samples from cases with influenza like illness 28% had received the influenza vaccine. This is consistent with our findings of 1/3 of children being vaccinated although the report mentions unpublished data of higher vaccination coverage rates reaching 40–60% between 2009–2011 in one large medical center in Beirut area and a rate of 6% across the country inferred from vaccine dose distribution [19]. These vaccination rates are much lower than what is estimated by the WHO for other childhood immunizations (above 85% for conjugate pneumococcal vaccine and above 90% for DTP, polio, Hib and hepatitis B. https://www.who.int/immunization/monitoring_surveillance/data/lbn.pdf) (as of July 6, 2020).

In this review awareness and previous encounters with influenza were the motivators for vaccination whereas fear of side effects, impression of ineffectiveness and lack of strong provider recommendation represented the major barriers to influenza vaccination in Lebanon [19]. This is consistent with our findings that reveal physician recommendation as the single most important factor in awareness and practice among Lebanese parents. This is consistent as well with other studies that show physician recommendation as the single most important player in influenza vaccine acceptance and uptake. Previous studies from developed and developing countries exploring parents attitudes towards influenza vaccine have in fact revealed that most parents are willing to administer influenza immunization to their children if recommended by their health care provider [10, 20–22]: a recent study from the US showed children who received provider recommendation for influenza vaccine were twice as likely to be vaccinated, however only 70% of individuals included in the study had received provider recommendation to receive the influenza vaccine [6]. In a study where 3 scenarios were presented to parents the scenario most likely to result in influenza vaccination (64% of cases) was recommendation by a physician [23].

Physician recommendation rates vary by country. In a study from Hong Kong only 10.6% of parents of children 6–23 months had received physician recommendation to give the influenza vaccine to their children [21]; a study from Japan showed low recommendation rates with only 19% of parents reporting having received physician recommendation to vaccinate their children against influenza [24]. In our study 47% of parents who did not vaccinate against influenza stated lack of physician recommendation as the reason. A study from Australia that investigated parental and PCP concerns about the flu vaccine showed that PCPs had concerns about efficacy, cost to families, morbidity due to influenza in otherwise healthy children [9]. Therefore, lack of recommendation by physician might reflect underlying hesitancy or lack in knowledge in physicians themselves, which need to be studied and addressed. Other studies have identified perceiving vaccination as effective [8, 10] and viewing their child as susceptible to disease [5, 8, 25], and having previously vaccinated their children [8, 23]. This is comparable to our findings showing that parents who vaccinated their children against influenza believed that vaccines prevent serious illness and protect others in the community from illness.

Vaccine hesitancy has also been reported to be associated with lower rates of vaccination. In the review of influenza vaccination in selected MENA countries, Lebanon was the only country out of 10 countries reviewed that lacked a national influenza immunization policy [19]. Presence of such a policy could empower physicians in recommending the influenza vaccine. As a matter of fact, in one study from Jordan incorporation of the influenza vaccine into the national immunization program was one of the key motivators for parents to administer influenza vaccine to their children in addition to physician recommendation [26]. Moreover, unlike other non-government funded vaccines, thus considered by the public not obligatory, the influenza vaccine is not listed in the current health record provide to all newborns by the MOPH although vaccines such as meningococcal vaccine, HPV vaccine and rotavirus vaccine have been added. Adding the influenza vaccine to the national health record could help also parents and providers see it as a more “legitimate” vaccine.

Current effort by MOPH including surveillance and online reporting of cases in addition to awareness campaigns directed to the public are a step forward in achieving better vaccination coverage rates; however based on our findings efforts geared towards physicians and public awareness utilizing TV, distribution of pamphlets and group discussion might have a higher yield in improving vaccination acceptance and uptake.

The study has some limitations including geographic limitation to the greater Beirut area, where influenza vaccine uptake might be higher than in rural areas. Vaccination rates in our population may thus be overestimated. A larger study including parents from different regions of the country would be more reflective of knowledge, attitudes and practice in the Lebanese population overall and would help devise national policies that would target and apply to the entire population. One third of vaccinated children in the private schools’ group were preschool children whereas public schools do not have preschool; this is a limitation in comparing between private and public schools. Moreover, the study only included children above 3 years of age, rates of vaccination in the younger population might be different.

On the other hand, the study has a good sample size and a high response rate; questionnaires were extensive covering knowledge, attitudes and practice and fully anonymous. This is one of few studies investigating knowledge, attitudes and practice towards influenza vaccine in a LMIC population and the first to investigate parental attitudes towards immunizing their children in Lebanon. A recent review of publications on seasonal influenza vaccine in the Eastern Mediterranean Region showed that this is an understudied topic in the region, especially when it comes to studies investigating uptake of vaccine in children and parental attitudes towards the vaccine. Our study should lead the way to further studies building on our findings and allowing to exploit them further into targeted policy building.

## Conclusion

Our study showed that one third of school aged children vaccinate against influenza: rates were lower in public schools when compared with private schools. Factors that influenced vaccination included child’s age, paternal employment and household income. Lack of vaccine recommendation by physician and hesitancy and poor trust in vaccination in general were associated with lower vaccination rates. Future larger studies at the national level are needed to confirm and generalize our findings. Our study suggests the need to investigate factors behind lack of physician recommendation of influenza vaccine improve awareness and recommendation amongst physicians as this was identified as one of the main drivers of immunization. Improving influenza vaccination rates may carry over to other currently available vaccines and potentially future vaccines covering other viruses responsible for seasonal or other epidemics.

## Supporting information

S1 Appendix Demographics and survey questionnaires (knowledge and believes, general attitude and trust, practice and behavior). (DOCX)

S2 Appendix Themes. (DOCX)

S3 Appendix Results by type of school. (DOCX)

S4 Appendix Survey questionnaires in Arabic. (DOCX)

S1 File Minimal dataset. (XLSX)
