# Supplementary material for: Knowledge, attitude and practice of influenza vaccination among Lebanese parents: A cross-sectional survey from a developing country
Source: PLoS One. 2021 Oct 14;16(10):e0258258. doi: 10.1371/journal.pone.0258258 (PMC8516244; doi:10.1371/journal.pone.0258258)
Supplement: S1 Appendix — (DOCX) [file pone.0258258.s001.docx]

**S1 Appendix**

1. **Demographics**

| **Child’s information** |
| --- |
| **Age :** Years Months |
| **Grade :** □ Preschool □ Elementary school □ Middle school □ Secondary school |
| **Gender :** □ M □ F |
| **Primary Nationality:** □Lebanese □ non Lebanese |
| **Child order :** □ 1^st^ □ 2^nd^ or more |

| **Parent's information** |
| --- |
| **Parent filling the questionnaire:** □ Father □ Mother |
| **Mother age (years)** □18-20 □ 20-30 □ 30-50 □ >50  **Employment:** □ Employed □ Self-employed □ Not employed  **What is the highest degree or level of school you have completed?**  □ No formal schooling □ Less than high school □ High school graduate  □ Technical school graduate □ University/college |
| **Father age(years)** □ 18-20 □ 20-30 □ 30-50 □ >50  **Employment:** □ Employed □ Self-employed □ Not employed  **What is the highest degree or level of school you have completed?**  □ No formal schooling □ Less than high school □ High school graduate  □ Technical school graduate □ University/college  **Household income:**  □ <1000$/month □ 1000-5000 $/month  □ >5000$/month □ No answer |

1. **Survey Questionnaire:**
   1. **Knowledge and Believes**

(Answers: strongly agree, agree, undecided, disagree, and strongly disagree)

1. Childhood vaccines are effective in protecting my child from serious disease.

2. Having my child vaccinated is important for the health of others in my community

3. It is better for my child to develop immunity by getting sick than by getting vaccinated*.*

4. New vaccines carry more risks than older vaccines.

5. I don’t mind having my child receive more than 5 types of vaccine in one visit (this could be one or two injections

6. My child is getting too many vaccines (10 to 15) during the first two years of life which may weaken his immune system.

7. Vaccines my cause the following: Learning disabilities, Autism, Diabetes, Sudden infant death syndrome, Other chronic diseases

8. Vaccines are not tested enough for safety.

9. Vaccines are given to children to prevent diseases that are not serious.

10. Vaccines make the immune system stronger.

11. Vaccination is for all ages, not only children.

12. No need for polio or measles vaccine anymore because of the eradication of these diseases.

13. There are situations in which you can't give live vaccines (ie: MMR,Varicella and OPV).

14. Harmful effects of vaccines are more than the benefits.

15. A healthy child does not need vaccination

16. There is a uniform immunization guideline for paediatric patients.

17. Lebanese parents are getting enough information about vaccines and their safety

18. The most common barrier(s) against vaccination in Lebanon is (are) (you can choose more than one): Lack of awareness; Financial issue; Fear; No barrier; Lack of access or availability of the vaccine

19. From where do you get the information you need about vaccines? (you can choose more than one): My Doctor; TV programs; Internet/Social Media; Friends; School; Others (Specify):

20. What do you think is the best way to raise awareness about vaccination? (you can choose more than one): Group meetings between parents and the paediatricians arranged by schools; Pamphlets from the MOH; Internet/Social Media; SMS from doctors or MOH; TV programs; Doctor offices; Others (Specify).

- 1. **General Attitude and Trust**

(Answers: strongly agree, agree, undecided, disagree, and strongly disagree)

21. The only reason I have my child get vaccinated is so that they can enter day-care or school.

22. I trust the information I receive about shots

23. There is an alternative way (hygiene or better nutrition) to protect my child from infection than vaccinating him.

1. I am satisfied with vaccination program offered by Ministry of Health.
2. I am satisfied with the way vaccines are given to my child when someone other than my pediatrician is injecting it. (i.e.: nurse, medical student, resident)

26. I have been often reluctant or hesitant to get a vaccination for my child.

27. I know parents who do not vaccinate their children because of religious or cultural reasons.

1. I am satisfied with my doctor’s answers to my questions related to immunization.
2. Generally I do what my doctor recommends about vaccines for my child/children.
3. I am concerned about serious adverse effects of vaccines.
4. I am concerned that newer vaccines are not as safe as older vaccines because they haven’t been tested or tracked for as long.
5. If you had another baby today, would you want him/her to get all the recommended shots?

(Yes, No, Undecided)

1. I want to know more about vaccination to help me ease my concerns. (Yes, No, Undecided)
2. When discussing the effects of vaccines with my doctor, I am concerned mostly about: (You can choose more than one) Fever; Rash; Diarrhea; Seizure; Site of injection infection; Other(Specify):
3. My greatest concern about childhood vaccines is/are: (you can choose more than one)

Too many vaccines given; Vaccines are not safe; Vaccines are not necessary; Vaccines cause diseases and long term complications; Vaccines might not prevent the disease; Vaccines cause side effects, such as fever and pain; No concerns; Other (specify)…

**Answers for questions 36-40: Always, frequently, rarely and never**

1. Overall, I consider myself hesitant about childhood vaccines.
2. I am in favor of vaccination.
3. I recommend vaccination to others.
4. I am concerned that childhood vaccines might not be safe. (might have a serious side effect )
5. I am concerned that a vaccine might not prevent the disease.
6. The maximum number of vaccine injections I am comfortable for my child to get per visit is:

1 to 2; 3 to 4; More than 4; Whatever the doctor recommends

- 1. **Practice and Behavior**

1. I have had to refuse or delay vaccination for my child in the past: Yes; No
2. If YES, please specify the cause (you can choose more than one answer)?

Did not think it was needed; Someone else told me that the vaccine was not safe; Did not know where to get vaccination; Heard or read negative media; Did not know where to get good; Did not think the vaccine was effective reliable information; Did not think the vaccine was safe/concerned; Fear of needles about side effects; Had a bad experience or reaction with previous vaccination; Religious reasons; Other (specify)…

1. My child has received vaccines as recommended per his/her pediatrician.

No; Yes, he/she received all; Yes, he/she received mandatory vaccines only

1. My child did receive the rotavirus vaccine: Yes; No
2. If NO, please specify the reason.

Missed the timeframe for giving it; It was too expensive at the time; Was hesitant and feared it might cause diarrhea; It was not recommended by my pediatrician; Other (specify)…

1. My child receives the flu vaccine almost every year: Yes; No .
2. If NO, please specify the reason

Missed the timeframe for giving it; It was too expensive at the time; Was hesitant and feared it might actually cause the flu; It was not recommended by my doctor; Getting the flu is not fatal; Other (specify)…

1. **I** vaccinated or will vaccinate my child against cervical cancer and cervical warts.

Yes; No; I am not aware of this vaccine

1. 1f No, please specify the reason

Missed the timeframe for giving it; It was too expensive at the time; Was hesitant that it is still a new vaccine and might have long term complications; It was not recommended by my doctor; Cultural/religious reasons; Too little known whether it is truly effective; Other (specify)…
