# Supplementary material for: Knowledge, attitude and practice of influenza vaccination among Lebanese parents: A cross-sectional survey from a developing country
Source: PLoS One. 2021 Oct 14;16(10):e0258258. doi: 10.1371/journal.pone.0258258 (PMC8516244; doi:10.1371/journal.pone.0258258)
Supplement: S2 Appendix — (DOCX) [file pone.0258258.s002.docx]

**S2 Appendix: Themes**

**Table 1: Knowledge and believes questions**

| **Efficacy** |
| --- |
| **Q1:** Childhood vaccines are effective in protecting my child from serious disease |
| **Q2:** Having my child vaccinated is important for the health of others in my community |
| **Q9:** Vaccines are given to children to prevent diseases that are not serious |
| **Q10:** Vaccines make the immune system stronger |
| **Safety** |
| **Q3:**  It is better for my child to develop immunity by getting sick than by getting vaccinated |
| **Q4:**  New vaccines carry more risks than older vaccines |
| **Q5:**  I don’t mind having my child receive more than 5 types of vaccine in one visit (this could be one or two injections) |
| **Q6:**  My child is getting too many vaccines (10 to 15) during the first two years of life which may weaken his immune system |
| **Q7a:** Vaccines may cause learning disabilities |
| **Q7b:**  Vaccines may cause autism |
| **Q7c:**  Vaccines may cause diabetes |
| **Q7d:**  Vaccines may cause sudden infant death syndrome |
| **Q7e:**  Vaccines may cause other chronic diseases |
| **Q8:**  Vaccines are not tested enough for safety |
| **Q14:**  Harmful effects of vaccines are more than the benefits |
| **General knowledge** |
| **Q11:**  Vaccination is for all ages, not only children. |
| **Q12:**  No need for polio or measles vaccine anymore because of the eradication of these diseases. |
| **Q13:**  There are situations in which you can't give live vaccines (ie: MMR,Varicella and OPV). |
| **Q15:**  A healthy child does not need vaccination. |
| **Q16:**  There is a uniform immunization guideline for pediatric patients. |
| **Q17:** Lebanese parents are getting enough information about vaccines and their safety. |

| **Reasons** |
| --- |
| **Q21:**  The only reason I have my child get vaccinated is so that they can enter day-care or school |
| **Q23:**  There is an alternative way (hygiene or better nutrition) to protect my child from infection than vaccinating him |
| **Q27:**  I know parents who do not vaccinate their because of religious or cultural reasons |
| **Trusts** |
| **Q22:**  I trust the information I receive about shots |
| **Q24:**  I am satisfied with vaccination program offered by Ministry of Health |
| **Q25:**  I am satisfied with the way vaccines are given to my child when someone other than my pediatrician is injecting it. ( i.e: nurse, medical student, resident..) |
| **Q28:**  I am satisfied with my doctor’s answers to my questions related to immunization |
| **Q29:**  Generally I do what my doctor recommends about vaccines for my child/children |
| **Q38:** I recommend vaccination to others |
| **Hesitancy** |
| **Q26:**  I have been often reluctant or hesitant to get a vaccination for my child |
| **Q30:**  I am concerned about serious adverse effects of vaccines |
| **Q31:**  I am concerned that newer vaccines are not as safe as older vaccines because they haven’t been tested or tracked for as long |
| **Q36:** Overall, I consider myself hesitant about childhood vaccines |
| **Q37**: I am in favor of vaccination |

**Table 2: General attitude and trust questions**

**Table 3: Practice and behavior questions.**

| **Practice Behavior** |
| --- |
| **Q42:** I have had to refuse or delay vaccination for my child in the past |
| **Q43:** If YES, please specify the cause (you can choose more than one answer):  Did not think it was needed  Did not know where to get vaccination  Did not know where to get good/reliable information  Did not think the vaccine was safe/concerned about side effects  Had a bad experience or reaction with previous vaccination  Someone else told me that the vaccine was not safe  Heard or read negative media  Did not think the vaccine was effective  Fear of needles  Religious reasons  Other (specify)… |
| 1. **Q44:** My child has received vaccines as recommended per his/her pediatrician |
| **Q47:** My child received the flu vaccine almost every year |
| **Q48:** If NO please specify the reason:  Missed the timeframe for giving it  It was too expensive at the time  Was hesitant and feared it might actually cause the flue  It was not recommended by my doctor  Getting the flu is not fatal  Other (specify)… |
