# Supplementary material for: Knowledge, attitude and practice of influenza vaccination among Lebanese parents: A cross-sectional survey from a developing country
Source: PLoS One. 2021 Oct 14;16(10):e0258258. doi: 10.1371/journal.pone.0258258 (PMC8516244; doi:10.1371/journal.pone.0258258)
Supplement: S3 Appendix — (DOCX) [file pone.0258258.s003.docx]

Table 1: Demographic characteristics in relation to Influenza vaccination in private and public schools

|  | **Private school** | | | **Public school** | | |
| --- | --- | --- | --- | --- | --- | --- |
|  | **No N=116 N.(%)** | **Yes N=56**  **N.(%)** | **p** | **No N=100**  **N.(%)** | **Yes N=34**  **N.(%)** | **p** |
| **Child Age, (Mean ± SD)** | 10.57 ± 3.54 | 8.85 ± 3.54 | 0.004 | 13.68 ± 1.26 | 13.35 ± 1.04 | 0.29 |
| **School Grade,** |  |  | 0.01 |  |  | 0.39 |
| Preschool | 11 (9.6) | 16 (28.6) |  | 0 (0.0) | 0 (0.0) |  |
| Elementary School | 63 (54.8) | 26 (46.4) |  | 2 (2.2) | 1 (2.9) |  |
| Middle School | 26 (22.6) | 12 (21.4) |  | 88 (94.6) | 30 (88.2) |  |
| Secondary School | 15 (13.0) | 2 (3.6) |  | 3 (3.2) | 3 (8.8) |  |
| **Parent filling the questionnaire,** | |  | 0.045 |  |  | 0.13 |
| Father | 24 (21.6) | 5 (9.1) |  | 14 (15.2) | 8 (27.6) |  |
| Mother | 87 (78.4) | 50 (90.9) |  | 78 (84.8) | 21 (72.4) |  |
| **Mother Age** |  |  | 0.11 |  |  | 0.89 |
| 18-20 | 0 (0.0) | 0 (0.0) |  | 2 (2.1) | 1 (3.1) |  |
| 20-30 | 2 (1.7) | 5 (8.9) |  | 4 (4.1) | 2 (6.3) |  |
| 30-50 | 107 (93.0) | 48 (85.7) |  | 86 (88.7) | 28 (87.5) |  |
| >50 | 6 (5.2) | 3 (5.4) |  | 5 (5.2) | 1 (3.1) |  |
| **Mother’s employment, n(%)** | |  | 0.92 |  |  | 0.96 |
| Employed | 75 (65.8) | 35 (62.5) |  | 8 (8.2) | 3 (10.0) |  |
| Self-Employed | 13 (11.4) | 7 (12.5) |  | 10 (10.3) | 3 (10.0) |  |
| Not Employed | 26 (22.8) | 14 (25.0) |  | 79 (81.4) | 24 (80.0) |  |
| **Mother’s Education** |  |  | 0.19 |  |  | 0.20 |
| No Formal Schooling | 1 (0.9) | 0 (0.0) |  | 4 (4.2) | 4 (13.3) |  |
| Less than High School | 4 (3.5) | 4 (7.3) |  | 38 (39.6) | 11 (36.7) |  |
| High School Graduate | 16 (13.9) | 2 (3.6) |  | 32 (33.3) | 9 (30.0) |  |
| Technical School/Graduate | 10 (8.7) | 5 (9.1) |  | 8 (8.3) | 0 (0.0) |  |
| University/College | 84 (73.0) | 44 (80.0) |  | 14 (14.6) | 6 (20.0) |  |
| **Father’s Age** |  |  | 1.00 |  |  | 0.02 |
| 18-20 | 0 (0.0) | 0 (0.0) |  | 1 (1.1) | 0 (0.0) |  |
| 20-30 | 0 (0.0) | 0 (0.0) |  | 2 (2.1) | 0 (0.0) |  |
| 30-50 | 90 (81.1) | 45 (81.8) |  | 59 (62.1) | 23 (74.2) |  |
| >50 | 21 (18.9) | 10 (18.2) |  | 33 (34.7) | 8 (25.8) |  |
| **Father’s Education** |  |  | 0.74 |  |  | 0.27 |
| No Formal Schooling | 1 (0.9) | 1 (1.9) |  | 8 (8.3) | 3 (10.0) |  |
| Less than High School | 10 (8.9) | 5 (9.3) |  | 45 (46.9) | 22 (36.7) |  |
| High School Graduate | 19 (17.0) | 7 (13.0) |  | 21 (21.9) | 12 (40.0) |  |
| Technical School Graduate | 6 (5.4) | 1 (1.9) |  | 10 (10.4) | 3 ()10.0 |  |
| University/College | 76 (67.9) | 40 (74.1) |  | 12 (12.5) | 1 (3.3) |  |
| **Father’s employment, n(%)** | |  | 0.35 |  |  | 0.02 |
| Employed | 79 (71.8) | 35 (64.8) |  | 55 (57.9) | 11 (34.4) |  |
| Self-Employed | 29 (26.4) | 19 (35.2) |  | 36 (37.9) | 21 (65.6) |  |
| Not Employed | 2 (1.8) | 0 (0.0) |  | 4 (4.2) | 0 (0.0) |  |
| **Household Income, n (%)** | |  | 0.002 |  |  | 0.82 |
| <1000$/month | 7 (8.3) | 4 (8.2) |  | 52 (75.4) | 14 (73.7) |  |
| 1000-5000$/month | 64 (76.2) | 24 (49.0) |  | 16 (23.2) | 5 (26.3) |  |
| >5000$/month | 13 (15.5) | 21 (42.9) |  | 1 (1.4) | 0 (0.0) |  |

Table 2: Parental knowledge regarding Immunization and its relation to influenza vaccine in private and public schools

|  | **Private school** | | | **Public school** | | |
| --- | --- | --- | --- | --- | --- | --- |
|  | **No N=116 N. (%)** | **Yes N=56**  **N. (%)** | **p** | **No N=100**  **N. (%)** | **Yes N=34**  **N (%)** | **p** |
| **Barriers** |  |  |  |  |  |  |
| Lack of awareness | 70 (61.9) | 36 (65.5) | 0.66 | 56 (57.1) | 17 (51.5) | 0.57 |
| Financial | 62 (54.9) | 40 (72.7) | **0.03** | 62 (63.3) | 14 (42.4) | **0.04** |
| No barriers | 20 (17.7) | 6 (10.9) | 0.25 | 14 (14.3) | 6 (18.2) | 0.59 |
| **Awareness** |  |  |  |  |  |  |
| Source of information  Doctor | 111 (95.7) | 54 (98.2) | 0.66 | 90 (94.7) | 33 (97.1) | 1.00 |
| TV | 18 (15.5) | 10 (17.9) | 0.70 | 23 (24.2) | 5 (14.7) | 0.25 |
| Internet | 29 (25.0) | 18 (32.1) | 0.33 | 19 (20.0) | 6 (17.6) | 0.77 |
| School | 11 (9.5) | 5 (8.9) | 0.91 | 25 (26.3) | 3 (8.8) | **0.03** |
| **Best way to raise awareness** | | 26 (46.4) | **0.02** | 58 (60.4) | 9 (55.9) | 0.64 |
| Group meeting | 32 (27.8) |  |  |  |  |  |
| Pamphlets | 47 (41.2) | 27 (48.2) | 0.39 | 41 (42.7) | 15 (44.1) | 0.89 |
| Internet | 39 (33.9) | 22 (39.3) | 0.49 | 23 (24.0) | 2 (5.9) | **0.02** |
| SMS | 22 (19.1) | 4 (7.1) | **0.04** | 29 (30.2) | 7 (20.6) | 0.28 |
| TV | 55 (47.8) | 25 (44.6) | 0.69 | 37 (38.5) | 5 (14.7) | **0.01** |
| Doctor | 66 (57.4) | 41 (73.2) | **0.045** | 50 (52.1) | 21 (61.8) | 0.33 |
| **Safety**  **Vaccines may cause** |  |  |  |  |  |  |

| a. Learning disabilities | 69.95 ± 21.72 | 77.40 ± 21.73 | 0.04 | 68.75 ± 21.91 | 74.22 ± 29.43 | 0.28 |
| --- | --- | --- | --- | --- | --- | --- |
| b. Autism | 66.51 ± 23.87 | 74.53 ± 24.75 | 0.05 | 73.49 ± 21.15 | 78.23 ± 23.93 | 0.31 |
| c. Diabetes | 69.86 ± 20.24 | 75.00 ± 23.00 | 0.15 | 70.83 ± 23.23 | 78.23 ± 24.78 | 0.14 |
| d. Sudden infant death syndrome | 68.92 ± 20.27 | 74.53 ± 21.08 | 0.11 | 66.37 ± 25.89 | 75.81 ± 27.75 | 0.09 |
| e. Other chronic diseases | 69.16 ± 20.48 | 73.11 ± 22.92 | 0.27 | 65.29 ± 26.47 | 74.19 ± 27.75 | 0.12 |
| **Efficacy** |  |  |  |  |  |  |
| **Efficacy score** | 74.80 ± 13.69 | 77.86 ± 11.93 | 0.15 | 73.33 ± 12.56 | 75.37 ± 10.65 | 0.40 |
| **Q1** Childhood vaccines are effective in protecting my child from serious disease. | 87.50 ± 17.36 | 94.44 ± 10.49 | 0.002 | 92.45 ± 12.63 | 92.65 ± 11.56 | 0.94 |
| **Q2**  Having my child vaccinated is important for the health of others in my community | 81.64 ± 22.41 | 91.51 ± 16.94 | 0.002 | 82.29 ± 21.43 | 80.15 ± 18.24 | 0.60 |
| **Q9** Vaccines are given to children to prevent diseases that are not serious. | 54.39 ± 27.07 | 47.32 ± 32.22 | 0.13 | 37.24 ± 32.03 | 40.91 ± 29.85 | 0.56 |
| **Q10** Vaccines make the immune system stronger | 75.66 ± 20.98 | 81.25 ± 20.37 | 0.10 | 82.47 ± 16.20 | 85.61 ± 14.02 | 0.32 |

Table 3: Association between attitude, trust and influenza vaccine in private and public schools

|  | **Private school** | | | **Public school** | | | |  |
| --- | --- | --- | --- | --- | --- | --- | --- | --- |
|  | **No N=116 N.(%)** | **Yes N=56**  **N.(%)** | **p** | | **No N=100**  **N.(%)** | **Yes N=34**  **N.(%)** | **p** | |
| **Perception of knowledge** | | | | | | | |  |
| Willingness to give Recommended shots | 91 (91.9) | 51 (98.1) | 0.16 | | 85 (96.6) | 30 (93.8) | 0.61 | |
| Concerns regarding vaccine | 91 (85.0) | 42 (82.4) | 0.66 | | 76 (88.4) | 29 (87.9) | 1.00 | |
| **Injection number** |  |  | 0.25 | |  |  | 0.91 | |
| 1 to 2 | 60 (52.2) | 22 (39.3) |  |  | 26 (27.7) | 9 (26.5) |  |  |
| 3 to 4 | 4 (3.5) | 4 (7.1) |  |  | 6 (6.4) | 1 (2.9) |  |  |
| More than 4 | 2 (1.7) | 0 (0.0) |  |  | 5 (5.3) | 23 (67.6) |  |  |
| Whatever the doctor recommends | 49 (42.6) | 30 (53.6) |  |  | 57 (60.6) | 23 (67.6) |  |  |
| **Concerns of the effects of vaccines** | | | | | | | |  |
| Fever | 92 (82.9) | 49 (87.5) | 0.44 | | 92 (93.9) | 28 (84.8) | 0.14 | |
| Rash | 24 (21.6) | 14 (25.0) | 0.62 | | 28 (28.6) | 5 (15.2) | 0.12 | |
| Diarrhea | 21 (18.9) | 9 (16.1) | 0.65 | | 31 (31.6) | 3 (9.1) | 0.01 | |
| Infection | 43 (38.7) | 19 (33.9) | 0.54 | | 45 (45.9) | 15 (45.5) | 0.96 | |
| Too numerous | 33 (28.7) | 12 (21.4) | 0.31 | | 16 (17.0) | 3 (9.1) | 0.40 | |
| Do not prevent disease | 24 (20.9) | 8 (14.3) | 0.30 | | 14 (15.1) | 8 (24.2) | 0.23 | |
| Side effects | 32 (27.8) | 24 (42.9) | 0.05 | | 58 (62.4) | 18 (54.5) | 0.43 | |
| No concern | 42 (36.5) | 21 (37.5) | 0.90 | | 38 (40.9) | 16 (48.5) | 0.45 | |
| **Trusts scores** | 65.41 ± 12.08 | 71.45 ± 11.20 | 0.002 | | 73.62 ± 10.34 | 75.64 ± 10.46 | 0.33 | |
| Physicians answers | 73.91 ± 18.55 | 81.25 ± 19.22 | 0.02 | | 81.38 ± 13.10 | 83.82 ± 17.28 | 0.45 | |
| Physicians recommendations | 79.82 ± 19.92 | 88.42 ± 16.62 | 0.01 | | 81.19 ± 16.15 | 85.61 ± 16.57 | 0.18 | |
| Recommend immunizations to others | 79.71 ± 27.47 | 89.28 ± 19.18 | 0.02 | | 93.47 ± 18.41 | 94.12 ± 12.90 | 0.85 | |
