# Supplementary material for: Knowledge, attitude and practice of influenza vaccination among Lebanese parents: A cross-sectional survey from a developing country
Source: PLoS One. 2021 Oct 14;16(10):e0258258. doi: 10.1371/journal.pone.0258258 (PMC8516244; doi:10.1371/journal.pone.0258258)
Supplement: S4 Appendix — (DOCX) [file pone.0258258.s004.docx]

حضرات المشاركون الأعزّاء

أنتم مدعوون للاشتراك في بحث يحمل عنوان **" معرفة و موقف وممارسة الآباء و الأمهات اللبنانيين بالنّسبة لمسألة تلقيح أطفالهم".**

تقوم بإجراء هذه الدّراسة الدّكتورة فرح فيتروني والدّكتورة لمى شرف الدّين من قسم طبّ الأطفال والمراهقين في المركز الطّبّي في الجامعة الأميركيّة في بيروت.

الهدف من مشروع هذا البحث هو:

- دراسة مدى اطّلاع الآباء اللبنانيّين على نظام تلقيح أطفالهم وموقفهم تجاهه بالإضافة إلى تأثير كلّ ذلك على خياراتهم في تحصين أطفالهم من خلال اللّقاحات.
- من الإيجابيات المتوقّعة من هذه الدّراسة رفع مستوى الوعي حول أهميّة اللّقاحات وأهميّة هذه اللّقاحات في الوقاية من بعض الأمراض.

 بلقاحات الأطفال لنتائج هذه الدّراسة أن توضّح بعض المفاهيم والممارسات الخاطئة المتعلّقة - من الممكن

و بالتّالي يمكن المساهمة في مساعدة أطبّاء الأطفال على التّركيز على هذه المسائل بهدف رفع مستوى تلقيح الأطفال وبالمقابل خفض مستوى الأمراض المحصّنة باللّقاحات عند الأطفال الّذين تتراوح أعمارهم بين 3 و 18 سنة.

- كما أنّه من الممكن لنتائج هذه الدّراسة أن تتيح الفرصة لخلق استراتيجيّة وضع خطّة توعية وسنّ قوانين حول لقاحات الأطفال.

**من أجل إنجاز هذا البحث، يرجى منكم ملء هذا البيان الاستقصائي بشكلٍ كامل**. تستغرق مدّة ملء هذا البيان الاستقصائي **١٠ دقائق من وقتكم**.

إنّ إنجازكم وتسليمكم لهذا البيان الاستقصائيّ يعني موافقتكم على المشاركة في هذه الدّراسة. مع العلم بانه لا يمكن من خلال هذا الاستبيان تحديد شخصيّة المشاركين بما أنّ الإجابات ستسجّل متجاهلة َهويّة المشترك.

وتجدر الإشارة إلى اتالي:

- أنهّ سيتمّ إشراك جميع أولياء التّلامذة اللّبنانيّين ( من عمر 3-18 سنة) والمسجّلين في المدارس المختارة في هذه الدّراسة في حال الموافقة على ملء الاستبيان. كما أنّه سيتمّ استبعاد كلّ أولياء التّلامذة الّذين لا يحملون الجنسيّة اللّبنانيّة كجنسيّة أولى من هذه الدّراسة.
- يحقّ فقط للأهل الّذين تفوق أعمارهم ال 18 سنة تعبئة هذه الاستمارة وبالتّالي المشاركة في هذه الدّراسة.

اشتراككم في هذا الاستبيان طوعيّ ولكم ملء الحريّة في الانسحاب من هذه الدّراسة في أيّ وقت ترغبون بذلك. نرجو الإشارة إلى أنّ اشتراككم الطّوعيّ لا يعود عليكم أو علينا بأيّ فائدة ماديّة أو معنويّة. كما نرجو إعلامكم بأنّ عدم رغبتكم بالمشاركة أو قراركم بالانسحاب من الدّراسة لا يرتّب عليكم أيّ بند جزائيّ أو خسارتكم لأيّ فائدة تحصلون عليها من أيّ من المؤسّسات المشمولة ضمن الدّراسة كمدرسة أولادكم والجامعة الأميركيّة ومركزها الطّبيّ ، كما لا تؤثّر على علاقتكم بهذه المؤسّسات.

لقد تمّت الموافقة على هذه الدّراسة من قبل لجنة الأخلاقيّات في الجامعة الأميركية في بيروت ولا يوجد أيّ خطر مرافق للمشاركة فيها. كما أنّ الإجابات على الاستمارة والنتائج سيتمّ مراقبتها والإشراف عليها من قبل اللّجنة نفسها مع الحرص على المحافظة على السّريّة التّامة بالنّسية للنّتائج و هويّة المشاركين. كما يمكنكم ، إذا وددتم، فصل نموذج الموافقة عن الاستبيان والاحتفاظ به لسجلاّتكم الخاصّة. الرّجاء وضع الاستمارة بعد ملئها في الظّرف المرافق ، لصقه، ومن ثمّ إرساله مع أولادكم إلى المدرسة ليتمّ جمع الإجابات من قبل فريق الدّراسة.

في حال وددتم طرح أيّ سؤال يتعلّق بهذه الدّراسة أو ببيان الاستقصاء، الرّجاء الاتصال بالدّكتورة فرح فيتروني على العنوان الإلكتروني الآتي: [ff25@aub.edu.lb](mailto:ff25@aub.edu.lb) أو من خلال طلب الرقم 1223 من سنترال الجامعة الأميركية (01-350000) أو بالمشرفة على الدّراسة الدّكتورة لمى شرف الدّين على عنوانها الإلكتروني الآتي: [lc12@aub.edu.lb](mailto:lc12@aub.edu.lb)l أو على الرقم الآتي ): 5874 01-350000-ext (

كما يمكن الإتصال بلجنة الأخلاقيات في الجامعة الأميريكية على الرقم- ext 5545): 01374374 (

أمّا إذا أردتم الاتّصال بشخص ليس له صلة مباشرة بالدّراسة بهدف الاستفسار، التّعبير عن الرّأي، تقديم شكوى ما حول الدّراسة ، للسّؤال عن حقوق المشارك أو للحصول على أيّ معلومات إضافيّة فالرّجاء الاتّصال بالسّيّدة نادين كمال في قسم لجنة الأخلاقيات في الجامعة الأميريكية على الرقم الآتي:

5445) (01-350000-ext

نحن في غاية الامتنان لمشاركتكم في هذه الدّراسة.

د. فرح فيتروني، طبيبة مقيمة في طب الأطفال، مستشفى الجامعة الأميركية

د. لمى شرف الدّين، أستاذة مشاركة في طب الأطفال و الخدج السريري، مستشفى الجامعة الأميركية

**أ-التّركيبة السّكّانيّة**

| **معلومات حول الطّفل** |
| --- |
| **العمر** : ----------- السّنة : ----------- الشّهر : ----------- |
| **الصف:** مرحلة الرّوضات مرحلة المدرسة الإبتدائيّة مرحلة المدرسة المتوسّطة  مرحلة المدرسة الثّانويّة |
| **الجنس:** ذكر أنثى |
| **الجنسيّة الأساسيّة:** لبنانيّة غير لبنانيّة |
| ترتيب الولد في المنزل: البكر الثّاني او أكثر |
| **معلومات حول الآباء** |
| **مَن من الوالدين يملأ هذا الاستبيان؟** الوالد الوالدة |
| **عمر الوالدة(بالسّنوات) -**18 20 سنة 20-30 سنة 30-50 سنة أكثر من 50 سنة  **عمل الوالدة:** موظّفة عمل حرّ لا تعمل  **ما هي أعلى شّهادة أو درجة تعليميّة تمّ إنجازها من قبل الوالدة ؟**  لا دراسة مدرسية دراسة ثانويّة دراسة جامعية  دراسة أقلّ من المرحلة الثّانوي دراسة مهنيّة أو تقنيّة |
| **عمر الوالد (بالسّنوات)**  -1820 سنة 20-30 سنة 30-50 سنة أكثر من 50 سنة  **عمل الوالد:** موظّف عمل حرّ لا يعمل  **ما هي أعلى شّهادة أو درجة تعليميّة تمّ إنجازها من قبل الوالد ؟**  لا دراسة مدرسية دراسة ثانويّة دراسة جامعية  دراسة أقلّ من المرحلة الثّانوي دراسة مهنيّة أو تقنيّة  **مدخول الأسرة الشّهريّ :**  أقلّ من 1000 $ شهريُّا 1000-5000 $ شهريًّا أكثر من 5000 $ شهريًّا لا إجابة |

*“الرجاء الإجابة على الأسئلة التالية باختياركم الإجابة الاكثر تعبيرا عن رأيكم - إجابة واحدة لكل سؤال"*

**ب- المعرفة والمعتقدات**

|  | أوافق بشدة | أوافق | على الحياد | أعارض | أعارض بشدة |
| --- | --- | --- | --- | --- | --- |
| 1. لقاحات الأطفال تحمي طفلي من الأمراض الخطيرة |  |  |  |  |  |
| 1. تلقيح طفلي مهمّ من أجل صحّة الآخرين في مجتمعي |  |  |  |  |  |
| 1. من الأفضل أن يحصل طفلي على المناعة من خلال إصابته بالمرض بدل الحصول عليه من خلال اللّقاح |  |  |  |  |  |
| 1. اللّقاحات الجديدة تحوي مخاطر أكثر من اللّقاحات القديمة |  |  |  |  |  |
| 1. لا أعارض تلقّي ولدي لأكثر من 5 أنواع من اللّقاحات أثناء زيارة واحدة للطّبيب (ممكن للّقاحات الخمس أن تكون من خلال حقنة واحدة أو اثنتين) |  |  |  |  |  |
| 1. يتلقّى ولدي الكثير من اللّقاحات   (10 – 15) خلال أوّل سنتين من حياته ممّا قد يضعف جهاز المناعة لديه |  |  |  |  |  |
| 7- ممكن للّقاحات أن تتسبّب في ما يلي: |  |  |  |  |  |
| أ - صعوبات تعلّميّة |  |  |  |  |  |
| ب- توحّد |  |  |  |  |  |
| ج- سكّري |  |  |  |  |  |
| د- الموت المفاجئ عند الأطفال |  |  |  |  |  |
| هـ - أمراض مزمنة أخرى |  |  |  |  |  |
| 8- لا تجرى اختبارات سلامة كافية للّقاحات |  |  |  |  |  |
|  | أوافق بشدة | أوافق | على الحياد | أعارض | أعارض بشدة |
| 9-تعطى اللقاحات للأطفال من أجل تحصينهم من الأمراض غير الخطرة |  |  |  |  |  |
| 10- تجعل اللّقاحات جهاز المناعة أقوى على الأمراض |  |  |  |  |  |
| 11- تستعمل اللّقاحات لجميع الفئات العمريّة وليس فقط للأطفال |  |  |  |  |  |
| 12- لا ضرورة للقاح شلل الأطفال والحصبة بسبب انقراض هذين المرضين. |  |  |  |  |  |
| 13- هناك حالات تمنع إعطاء لقاحات تحتوي على جراثيم أو فيروسات حيّة كلقاحات شلل الأطفال، جدرة الماء واللّقاح الثّلاثي |  |  |  |  |  |
| 14- ضرر اللّقاحات أكبر من فوائدها |  |  |  |  |  |
| 15- الطّفل الّذي يتمتّع بصحّة جيّدة لا يحتاج للّقاحات |  |  |  |  |  |
| 16-للّقاحات عند الأطفال مبادئ وأصول توجيهيّة موحّدة عالميَّا |  |  |  |  |  |
| 17- ينال الأهل في لبنان معلومات كافية حول اللّقاحات وسلامتها |  |  |  |  |  |
| 18- **أبرز المعوقات الّتي تقف حائلاً ضدّ إعطاء اللّقاحات في لبنان هي: ( بامكانكم اختيار أكثر من إجابة واحدة)**  نقص في التّوعية المسائل الماديّة الخوف لا معوّقات  عدم توفر اللقاح او امكانية الحصول عليه | | | | | |
| 19- **من أين تحصلون على المعلومات الّتي تحتاجون إليها حول اللّقاحات ؟ ( يمكنكم اختيار أكثر من إجابة واحدة)**  من طبيبي من البرامج التّلفزيونيّة من الانترنيت/ من وسائل التّواصل الاجتماعي  من الأصدقاء من المدرسة من مصادر أخرى (حدّد) | | | | | |
| 20- **برأيكم، ما هي أفضل الوسائل الّتي يجب اعتمادها للتّوعية على أهميّة اللّقاحات ؟ (يمكنكم اختيار أكثر من إجابة واحدة)**  لقاءات عامّة بين الأهل وأطبّاء الأطفال تنظّمها المدارس  كتيّبات توجيهيّة تصدرها وزارة الصّحّة الانترنيت ووسائل التّواصل الاجتماعي  رسائل نصّيّة يرسلها أطبّاء أو وزارة الصّحّة  برامج تلفزيونيّة عيادات الأطبّاء وسائل أخرى (حدّد) | | | | | |

*“الرجاء الإجابة على الأسئلة التالية باختياركم الإجابة الاكثر تعبيرا عن رأيكم - إجابة واحدة لكل سؤال"*

**ج- الموقف العام والثّقة**

|  | أوافق بشدة | أوافق | على الحياد | أعارض | أعارض بشدة |
| --- | --- | --- | --- | --- | --- |
| 21- السّبب الوحيد الّذي يدفعني لتلقيح طفلي هو تمكينه من الدّخول إلى الحضانة أو المدرسة |  |  |  |  |  |
| 22- أثق بالمعلومات الّتي أحصل عليها حول اللّقاحات |  |  |  |  |  |
| 23- يوجد طريقة بديلة عن اللّقاح لحماية طفلي من الأمراض وهي النّظافة العامّة والنّظام الغذائي السّليم |  |  |  |  |  |
| 24**-** أنا راضِ ببرنامج لقاحات وزارة الصّحة العامّة |  |  |  |  |  |
| 25- أنا راضِ عن الطّريقة الّتي تعطى فيها اللّقاحات عندما يقوم بذلك شخص آخر غير طبيب أطفالي الخاصّ (مثلاَ: الممرضة، تلميذ الطبّ، الطّبيب المقيم) |  |  |  |  |  |
|  | أوافق بشدة | أوافق | على الحياد | أعارض | أعارض بشدة |
| 26- أحيانّا كثيرة أكون متردّد/ متردّدة في إعطاء اللّقاح لطفلي |  |  |  |  |  |
| 27 - أعرف أهل لا يلقّحون أطفالهم لأسباب دينيّة، عقائديّة أو ثقافيّة |  |  |  |  |  |
| 28 - أنا راضية عن الإجابات الّتي أنالها من طبيب أطفالي عن أسئلتي حول اللّقاحات |  |  |  |  |  |
| 29 - عادة ألتزم بتوجيهات طبيب أطفالي في ما يتعلّق بلقاحات طفلي / أطفالي |  |  |  |  |  |
| 30- أنا قلق/ قلقة للآثار الجانبيّة الّتي قد تسبّبها اللّقاحات |  |  |  |  |  |
| 31- أنا قلق/ قلقة عندما يتعلّق الأمر بأيّ لقاح جديد خوفًا من آثاره الجانبيّة وعدم فعاليّته كاللّقاحات القديمة وذلك لقلّة التّجارب عليه وتتبّع مساره بشكل كافِ |  |  |  |  |  |
| **32 - إذا رزقتم بطفل آخر اليوم هل تريدون حقنه بكل اللّقاحات الموصى بها**  لا أدري كلا نعم | | | | | |
| **33- أريد أن أنال معلومات أوفى عن اللّقاحات كي تقلّ مخاوفي**  لا أدري كلا نعم | | | | | |
| **34-عند مناقشة موضوع اللّقاحات مع طبيبي، تتركّز مخاوفي حول:** (يمكنكم اختيار أكثر من إجابة واحدة)  ارتفاع الحرارة الطّفح الجلديّ الإسهال  نوبات الصّرع التهاب منطقة الحقنة أسباب أخرى ( حدّد) | | | | | |
| **35 - أكبر مخاوفي المتعلّقة بلقاحات الأطفال هي:** (يمكنكم اختيار أكثر من إجابة واحدة )  العدد الكبير للّقاحات الّتي تعطى للأطفال اللّقاحات ليست آمنة اللّقاحات ليست ضروريّة  تسبّب الّلقاحات أمراضا ومضاعفات طويلة الأمد ممكن للّقاحات ألاّ تحمي من المرض  اللّقاحات آثار جانبيّة كالحرارة والشّعور بالألم لا مخاوف عندي حول اللّقاحات  أسباب أخرى (حدّد) | | | | | |
| **36- بشكل عام، هل تصنّفون أنفسكم من المتردّدين في ما يتعلّق بموضوع اللّقاحات.**  دائماً معظم الأحيان نادراً أبداً | | | | | |
| **37 - أنا مع موضوع التّلقيح**  دائماً معظم الأحيان نادراً أبداً | | | | | |
| **38- أنا أنصح الآخرين بأخذ اللّقاحات**  دائماً معظم الأحيان نادراً أبداً | | | | | |
| **39- أنا قلق/قلقة من أن تكون لقاحات الأطفال غير آمنة ( لها آثار جانبيّة خطيرة )**  دائماً معظم الأحيان نادراً أبداً | | | | | |
| **40**- **أنا قلق/قلقة من أن لا تقي اللقاحات من المرض**  دائماً معظم الأحيان نادراً أبداً | | | | | |
| **41**- **لعدد الأمثل للقاحات الّتي أعتقد أنّ على طفلي أخذها خلال زيارة واحدة للطّبيب هي:**  من 1 الى 2 من 3 الى 4 أكثر من 4 لا فرق طالما أنه ما يوصي به الطبيب | | | | | |

*“الرجاء الإجابة على الأسئلة التالية باختياركم الإجابة الاكثر تعبيرا عن رأيكم - إجابة واحدة لكل سؤال"*

**د- الممارسة والسّلوك**

**42 -كان عليّ أن أرفض أو أؤخّر تلقيح طفلي في الماضي**

نعم كلاّ لا ادري

**43- إذا كانت الإجابة نعم ، الرّجاء تحديد السّبب (يمكنكم اختيار أكثر من إجابة واحدة)**

| لم اعتقد بأنّ اللّقاح كان ضروريَّا | أحدهم أخبرني أنّ اللّقاح لم يكن آمنَا |
| --- | --- |
| كنت أجهل المكان الّذي يعطى فيه اللّقاح | سمعت أو قرأت معلومات سلبيّة عن اللّقاح |
| كنت أجهل من أين أحصل على معلومات جيّدة وموثوقة تتعلّق باللّقاح | لم أكن أعتقد أنّ اللّقاح فعّال |
| لم أكن أعتقد أنّ اللّقاح آمن ولا آثار جانبيّة له | الخوف من الحقن |
| كان لي تجربة سيّئة سابقًا مع الّلقاحات وآثارها الجانبيّة | أسباب دينيّة |
| أسباب دينيّة |  |

**44-لقد حصل طفلي على اللّقاحات الّتي اوصى بها طبيب الأطفال الخاصّ به**

كلاّ نعم ، حصل/ حصلت عليها كلّها

نعم ،حصل/ حصلت على اللّقاحات الإلزاميّة فقط لا ادري او لا اتذكر

**45- لقد حصل طفلي على لقاح فيروس الرّوتا**

نعم كلاّ لا ادري او لا اتذكر

**45- إذا كانت الإجابة كلاّ الرّجاء تحديد السّبب**

| تخطّي الإطار الزّمنيّ المحدّد للتّلقيح | السّعر المرتفع للّقاح في ذلك الوقت |
| --- | --- |
| الخوف من أن يسبّب هذا اللّقاح الإسهال | لم يوص طبيب أطفالي بهذا اللّقاح |
| أسباب أخرى (حدّد) |  |

**47- يحصل طفلي على لقاح الكريب ( الإنفلونزا) سنويَّا**

نعم كلاّ لا ادري او لا اتذكر

**48- إذا كانت الإجابة كلاّ الرّجاء تحديد السّبب**

| تخطّي الإطار الزّمنيّ المحدّد للتّلقيح | الخوف من أن يسبّب هذا اللّقاح االكريب لطفلي |
| --- | --- |
| السّعر المرتفع للّقاح في ذلك الوقت | لم يوص طبيب أطفالي بهذا اللّقاح |
| أسباب أخرى (حدّد) |  |

49**- لقد لقّحت طفلي أو سألقحه ضدّ سرطان عنق الرّحم أو ثآليل عنق الرّحم**

نعم كلاّ لا ادري او لا اتذكر ليس لدي فكرة عن هذا اللقاح

50- **إذا كانت الإجابة كلاّ الرّجاء تحديد السّبب**

| تخطّي الإطار الزّمنيّ المحدّد للتّلقيح | السّعر المرتفع للّقاح في ذلك الوقت |
| --- | --- |
| الخوف من أن اللّقاح حديث ويمكن أن يكون له مضاعفات خطيرة في المستقبل | لم يوص طبيب أطفالي بهذا اللّقاح |
| لأسباب ثقافيّة أو دينيّة | المعلومات ضئيلة حول مدى فعاليّة هذا اللّقاح |
| أسباب أخرى (تحدّد) |  |
